# Multi-Omics Insights into the Role of Dulcitol in Weaned Piglets’ Growth Performance and Intestinal Health

**DOI:** 10.3390/antiox14111346

**Published:** 2025-11-10

**Authors:** Zhengqun Liu, Ning Li, Han Wang, Ruqing Zhong, Shanchuan Cao, Zi Zheng, Jingbo Liu, Liang Chen, Jun Yan, Shuqin Mu

**Affiliations:** 1 Tianjin Key Laboratory of Animal Molecular Breeding and Biotechnology, Tianjin Engineering Research Center of Animal Healthy Farming, Institute of Animal Science and Veterinary, Tianjin Academy of Agricultural Sciences, Tianjin 300381, China; liuzhengqun@foxmail.com (Z.L.);; 2 Tianfu Institute of Research and Innovation, Southwest University of Science and Technology, Chengdu 610299, China; 3 State Key Laboratory of Animal Nutrition and Feeding, Institute of Animal Science, Chinese Academy of Agricultural Sciences, Beijing 100193, China; 4 College of Life Science and Agri-Forestry, Southwest University of Science and Technology, Mianyang 621010, China

**Keywords:** dulcitol, growth performance, intestinal barrier, multi-omics, gut microbiota, weaned piglets

## Abstract

Weaning is known to induce oxidative stress and dysregulated inflammatory responses, thus damaging performance growth. This research was conducted to investigate the effects of dulcitol (Dul) on the growth performance and gut health of weaned piglets using multi-omics technologies. Two groups (*n* = 6, 6 pigs per replicate) of piglets (28 days old, BW, 8.91 ± 0.18 kg) were randomly assigned to receive either a basal diet supplemented with Dul (500 mg/kg) or without it for a period of 28 days. The findings indicated that the addition of Dul to the diet improved growth performance and had positive effects on antioxidant and anti-inflammatory statuses in weaned piglets (*p* < 0.05). Dul also strengthened intestinal barrier integrity via decreased diamine oxidase and D-lactate and elevated tight junction proteins (i.e., ZO-1, CLDN, and OCLN, *p* < 0.05). Multi-omics analyses demonstrated that Dul induced modifications in colonic protein expression associated with oxidative stress and glucose metabolism, altered linoleic acid metabolic pathways, and restructured the gut microbiota. This restructuring was characterized by a decreased prevalence of genera linked to inflammation and oxidative stress, including *Proteobacteria*, *Prevotella*, and *Prevotellaceae_NK3B31_group*. Collectively, the findings indicate that Dul promotes intestinal wellness and growth in weaned piglets through intricate interactions between gut microbiota and host metabolic processes.

## 1. Introduction

Weaning represents a significant and stressful transition in the developmental stages of piglets. The stress associated with weaning can lead to decreased feed consumption, impair intestinal barrier functions, provoke diarrhea, reduce overall growth, and may even result in mortality among piglets, which can ultimately incur economic losses [1]. Consequently, it is vital to identify strategies to mitigate weaning stress in weaned piglets for the benefit of animal husbandry.

Previous studies have shown that the stress of weaning can trigger oxidative stress and disrupt inflammatory responses, causing harm to the growth of piglets [2]. Furthermore, weaning stress has negative effects on intestinal function, including increased epithelial permeability, impaired intestinal morphology, and altered gut microbiota in piglets [3]. Previous studies showed that dietary supplementation with derivatives from natural products has the potential to alleviate weaning stress in piglets. Dulcitol (Dul), which is also referred to as galactitol, is derived from euonymus alatus and has been extensively utilized as a herbal remedy in Asia [4]. Reports indicate that Dul has various beneficial properties, including anti-cancer and anti-inflammatory effects [4,5,6]. Furthermore, our previous investigation demonstrated that Dul improved anti-inflammatory and antioxidant capabilities along with growth performance in growing-finishing pigs [7]. However, the effects of Dul on the growth performance of weaned piglets and the mechanisms behind it remain unclear. Proteomics is the large-scale study of proteins, particularly their structures, functions, and dynamics. Metabolomics is the comprehensive study of small molecule metabolites, which are the end products of cellular regulatory processes. Therefore, proteomics and metabolomics provide direct insight into the functional phenotype [8].

Thus, this study seeks to assess the effects of Dul on growth performance as well as on antioxidant and anti-inflammatory capacities in weaned piglets. Subsequently, we conducted metabolomics, proteomics, and gut microbial composition analysis, aiming to further explore the underlying mechanisms.

## 2. Materials and Methods

### 2.1. Animals and Treatments

A total of 72 weaned male piglets (Landrace × Yorkshire, 28 days old) with comparable body weight (BW, 8.91 ± 0.18 kg) were randomly divided into 2 groups (6 replicate pens (2 × 2 m) and 6 pigs per pen). The control group (Con) received a basal diet, while the Dul treatment group (Dul) was provided with a basal diet supplemented by 500 mg/kg Dul (obtained from Zhongwei International Life Technology Co., Ltd., Tianjin, China, with a purity of 99.88%). The basal diet was formulated mainly using corn-soybean meal (Supplementary Table S1), in accordance with the requirements set by the National Research Council [9]. All piglets were kept in a suitable environment (natural lighting, ambient temperature 25 ± 2 °C and relative humidity 55–65%), with continuous access to feed and water. The duration of the experiment was 28 days. At the end of the experiment, the piglets underwent a fasting period of 12 h before being weighed with replicate pens to determine their average daily gain (ADG), average daily feed intake (ADFI), and feed-to-gain ratio (F:G).

### 2.2. Serum Parameters Analysis

Blood samples (5 mL) were collected from each piglet via venipuncture of the anterior vena cava using tubes without anticoagulant. After allowing the samples to stand for 1.5 h, they were subsequently centrifuged at 3000× *g* and at a temperature of 4 °C for a duration of 15 min. Following this process, the concentrations of specific cytokines, namely IL-1beta (H002-1-2) and IL-10 (H009-1-2), were quantified using enzyme-linked immunosorbent assay (ELISA) kits from Nanjing Jiancheng Bioengineering Institute, adhering strictly to the manufacturer’s guidelines. Furthermore, various serum parameters were assessed, including the activities of superoxide dismutase (SOD, A001-3-2) and diamine oxidase (DAO, A088-3-1), along with the levels of malondialdehyde (MDA, A003-1-2) and D-lactate (D-lac, A019-3-1). These analyses were conducted using commercial assay kits from the same institute, following the manufacturer’s instructions meticulously, and ELISA analysis was performed following the manufacturer’s protocols in technical duplicates (*n* = 3).

### 2.3. Colon Morphology and Goblet Cell Analysis

Following blood collection, piglets were euthanized by intravenous injection of sodium pentobarbital (100 mg/kg body weight) via the ear vein, following the AVMA Guidelines for the Euthanasia of Animals (2020 edition). Subsequently, 1 cm segments from the midsection of the colon were aseptically excised and preserved in a 4% paraformaldehyde solution. Semi-serial 5 µm cross-sections were obtained with a Lupetec™ MRP-03 rotary microtome (São Carlos, São Paulo state, Brazil), mounted on histological slides, and deparaffinized in a drying oven. The colonic mucosa was meticulously scraped using glass slides, transferred into 2-mL sterile tubes, rapidly frozen in liquid nitrogen, and stored at −80 °C for targeted metabolomic analyses, protein identification, and sequencing of microbial 16S rRNA genes. The colonic goblet cells were stained with periodic acid-Schiff (PAS) according to the methods described in our previous study [10]. Briefly, deparaffinized colon segment slides were treated with 3% acetic acid and then stained with 1% PAS solution and sodium metabisulfite. Photomicrographs were captured using a Leica DM2000 light microscope with 50 × magnification (Leica Microsystems, Wetzlar, Germany). The crypt depth (CD) and the quantity of goblet cells were quantitatively determined by performing random measurements on six well-oriented and intact crypts.

### 2.4. Western Blot Analysis

To assess the total protein content in the colonic mucosa, the tissues were subjected to extraction utilizing RIPA lysis buffer. The protein concentration was subsequently quantified employing a bicinchoninic acid (BCA) protein assay kit from Thermo Fisher Scientific, located in Waltham, MA, USA. Equal amounts (30 μL) of the extracted proteins were then separated using sodium dodecyl sulfate polyacrylamide gel electrophoresis (SDS-PAGE). Following this, the proteins were transferred onto polyvinylidene difluoride (PVDF) membranes. In order to prevent non-specific binding, the membranes were blocked with a 5% skim milk solution at room temperature prior to being incubated 2 h with primary beta Actin (β-actin, bs-0061R, 1:5000, Bioss), Claudin-1 (CLDN, bs-42307R, 1:1000, Bioss), Zona occludens protein 1 (ZO-1, bsm-42209R 1:1000, Bioss, Beijing, China), and Occludin 1 (OCLN, bs-10011R, 1:1000, Bioss) at 4 °C. After thorough washing, the membranes were exposed to secondary antibodies at room temperature (6 times). Finally, the protein bands were visualized, and analysis was conducted with the aid of a Bio-Rad gel imaging system, ensuring that the relative protein levels of the target proteins were normalized against a reference protein for accurate comparison. Image J Program Analyzer (Media Cybernetics, Bethesda, MD, USA) was used to quantify the protein band density.

### 2.5. Proteomics Analysis

Proteins were extracted from the colonic mucosa (approximately 30 mg) using an SDT buffer comprising 4% sodium dodecyl sulfate (SDS), 100 mM dithiothreitol (DTT), and 150 mM Tris-HCl (pH 8.0). Each sample’s protein concentration was determined using a BCA Assay Kit provided by Bio-Rad (Hercules, CA, USA), following the manufacturer’s protocol. After quantification with BCA assay, 100 μg of protein per sample was digested with trypsin (Promega, sequencing grade) at an enzyme-to-protein ratio of 1:50 (*w*/*w*) in 100 mM TEAB buffer (pH 8.5). Digestion was performed at 37 °C for 16 h, followed by termination with 1% formic acid. The peptide samples were desalted through C18 cartridges (Empore™ SPE Cartridges, light 7 mm, with a volume of 3 mL, Sigma, St. Louis, MO, USA), and subsequently concentrated via vacuum centrifugation and reconstituted in formic acid. The peptide concentration was assessed by measuring the UV light spectral density at 280 nm before the peptide mixture was loaded onto an EASY-Spray™ C18 trap column. For LC-MS/MS analysis, the samples were separated on an EASY-Spray™ C18 column (75 μm × 25 cm, 2 μm particles) with a 120-min linear gradient of buffer B (80% acetonitrile, 0.1% formic acid) at 300 nL/min. The eluted peptides were then analyzed on a Q-Exactive HF-X mass spectrometer in data-independent acquisition (DIA) mode, with the ion source parameters set at 2.0 kV spray voltage and 320 °C capillary temperature. All procedures were performed according to standardized protocols provided by Shanghai Applied Protein Technology Co., Ltd., which are publicly available upon request. Raw data were processed using Spectronaut (v16.0, Biognosys) with default settings against the Sus scrofa UniProt database (UP000008227). Peptide and protein identifications were filtered at 1% FDR. Proteins with ≥2 unique peptides and fold-change > 1.5 with *p* < 0.05 were considered significantly altered.

### 2.6. Untargeted Metabolism Analysis

Metabolomics profiling of colonic content was determined by Shanghai Applied Protein Technology Co., Ltd. (Shanghai, China). Briefly, approximately 50 mg of colonic content sample was mixed with 400 μL of pre-cooled extraction solvent (methanol:acetonitrile:water = 2:2:1, *v*/*v*/*v*) and vortexed for 30 min. The samples underwent centrifugation at a force of 14,000× *g* for a duration of 20 min. Following this process, the samples were dried and subsequently filtered using a SCAA-104 filter (Thermo Fisher Scientific, Waltham, MA, USA) with a pore size of 0.22 μm. The filtered samples were then dissolved in a solvent mixture of acetonitrile and water at a ratio of 1:1 (*v*/*v*) to prepare for liquid chromatography-mass spectrometry (LC-MS) analysis. The mass spectrometer (Triple TOF 6600, Sciex, Framingham, MA, USA) was operated with an electrospray ionization source in both positive and negative modes. The specific parameters were: ion source gas 1: 60 psi, gas 2: 60 psi, curtain gas: 35 psi, source temperature: 600 °C, ion spray voltage floating: ±5500 V, collision energy: 10 eV, declustering potential: 60 V. Chromatographic separation of the components was achieved using a UPLC system that featured an ACQUITY UPLC CSH C18 column, measuring 150 × 2.1 mm with a particle size of 1.7 µm, manufactured by Waters Corporation (Milford, MA, USA). Once the separation was completed, the samples underwent analysis with a Triple TOF 6600 Mass Spectrometer from Agilent Technologies (Santa Clara, CA, USA), which was equipped with an electrospray ionization source capable of operating in both positive and negative ion modes. All procedures were performed according to standardized protocols provided by Shanghai Applied Protein Technology Co., Ltd., which are publicly available upon request. The raw data collected from this analysis was imported into Progenesis QI version 2.3 for the purposes of peak detection and alignment. Metabolites that exhibited a variable importance in projection (VIP) value greater than 1 and a *p* value of less than 0.05 were identified as significantly different metabolites. Metabolite identification was performed by matching the accurate mass and MS/MS spectra against the HMDB (Human Metabolome Database) and Metlin databases. The confidence level of identification followed the Metabolomics Standards Initiative (MSI) guidelines, with most identifications at level 2 (putatively annotated compounds).

### 2.7. Gut Microbiota Analysis

Total genomic DNA was extracted from the colonic mucosa utilizing a DNA extraction kit from Omega Bio-Tek (Omega Bio-tek, Norcross, GA, USA, M5625-01). DNA quality was assessed using NanoDrop 2000 (Thermo Fisher Scientific, Waltham, MA, USA) for A260/A280 ratio (acceptable range: 1.8–2.0) and 1% agarose gel electrophoresis to confirm integrity. The concentration and purity of the extracted DNA were assessed using 1% agarose gel electrophoresis. Amplification of the V3-V4 regions of the bacterial 16S rRNA gene was performed using gene-specific primers, namely 338F (5′-ACTCCTACGGGAGGCAGCAG-3′) and 806R (5′-GGACTACHVGGGTWTCTAAT-3′). PCR amplification was performed with 30 cycles: denaturation at 95 °C for 30 s, annealing at 55 °C for 30 s, extension at 72 °C for 45 s. Negative controls (no template) and positive controls (standard bacterial DNA) were included in each run. The amplification and subsequent sequencing were carried out following the standard protocols provided by Shanghai Applied Protein Technology in Shanghai, China, using the Illumina MiSeq platform (Illumina Inc., located in San Diego, CA, USA) with 2 × 250 bp paired-end reads. Average sequencing depth was 50,000 reads per sample. All procedures were performed according to standardized protocols provided by Shanghai Applied Protein Technology Co., Ltd., which are publicly available upon request. The resultant sequences were subjected to analysis, where they were categorized into operational taxonomic units (OTUs) based on a 97% similarity threshold.

### 2.8. Data Analysis

Statistical analyses were executed using the SAS PROC general linear model procedures (version 9.4, SAS Institute, Cary, NC, USA) with T test model, and the normality of all data, including alpha-diversity indices and serum parameters, was checked using the Shapiro–Wilk test. Data are expressed as the mean ± SEM. *p* < 0.05 was considered statistically significant.

For proteomics and metabolomics data, multivariate statistical analysis, including Principal Component Analysis (PCA) and Partial Least Squares-Discriminant Analysis (PLS-DA), was performed using MetaboAnalyst 5.0. Differential proteins/metabolites were identified based on a variable importance in projection (VIP) > 1.0 from the PLS-DA model and a *p*-value < 0.05 from a *t*-test. Pathway enrichment was analysed by Kyoto Encyclopedia of Genes and Genomes (KEGG) (www.genome.jp/kegg/ (accessed on 20 November 2024)) and Gene Ontology (GO; Gene Ontology Consortium, geneontology.org), and enrichment analysis was performed on differentially expressed proteins/metabolites using Fisher’s Exact Test. For gut microbiota, alpha-diversity, Shannon, Simpson’s evenness indexes and Goods coverage were calculated at 97% identity, and Student’s T test was used to assess significant differences between the CON and DUL groups. Beta-diversity was investigated with QIIME using principal coordinate analysis (PCoA) based on the weighted Unifrac distance matrix, and permutational multivariate analysis of variance (PERMANOVA) with the adonis function was calculated to determine significant differences in the microbial community.

## 3. Results

### 3.1. Dul Improved Growth Performance in Weaned Piglets

**Figure 1:**
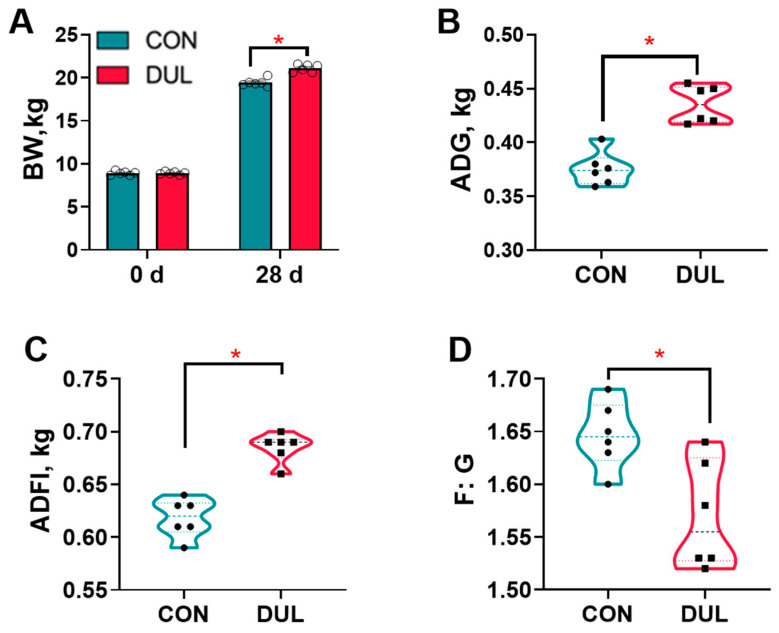
Dul improved the growth performance of weaned piglets (*n* = 6). (**A**) BW; (**B**) ADG; (**C**) ADFI; (**D**) F:G. *n* = 6 per group, * *p* < 0.05.

Throughout the entire duration of the study, all the piglets exhibited a healthy condition, which is a notable observation. The data on growth performance is illustrated in Figure 1. The administration of Dul treatment led to a significant enhancement in BW, ADG, and ADFI among the piglets when compared to the control group, as depicted in Figure 1A–C (*p* < 0.05). Furthermore, it was observed that the inclusion of Dul in the diet notably decreased the F:G for the piglets, as shown in Figure 1D (*p* < 0.05).

### 3.2. Dul Enhanced Antioxidant and Anti-Inflammatory Capacities in Weaned Piglets

**Figure 2:**
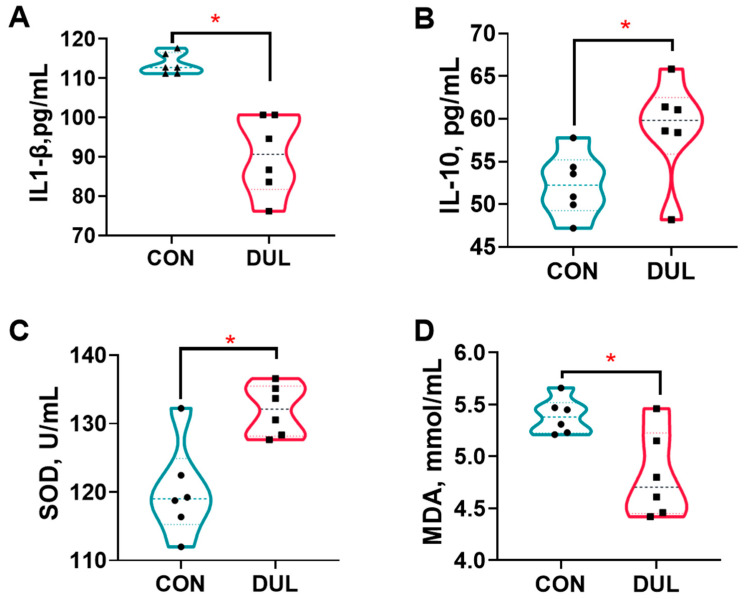
Dul enhanced the anti-oxidant and anti-inflammatory capacities of weaned piglets (*n* = 6). (**A**) Serum level of IL-1beta; (**B**) Serum level of IL-10; (**C**) serum activity of SOD; (**D**) Serum level of MDA. *n* = 6 per group, * *p* < 0.05.

The results indicated that Dul treatment resulted in a significant reduction in serum levels of interleukin-1 beta (IL-1β), while simultaneously increasing the serum levels of interleukin-10 (IL-10) in the piglets, as represented in Figure 2A,B (*p* < 0.05). In addition to changes in cytokine levels, Dul supplementation was found to enhance the activity of SOD and MDA levels in the serum of the piglets, as illustrated in Figure 2C,D (*p* < 0.05).

### 3.3. Dul Decreased Intestinal Permeability but Increased the Expression of Colonic Tight Junction in Weaned Piglets

**Figure 3:**
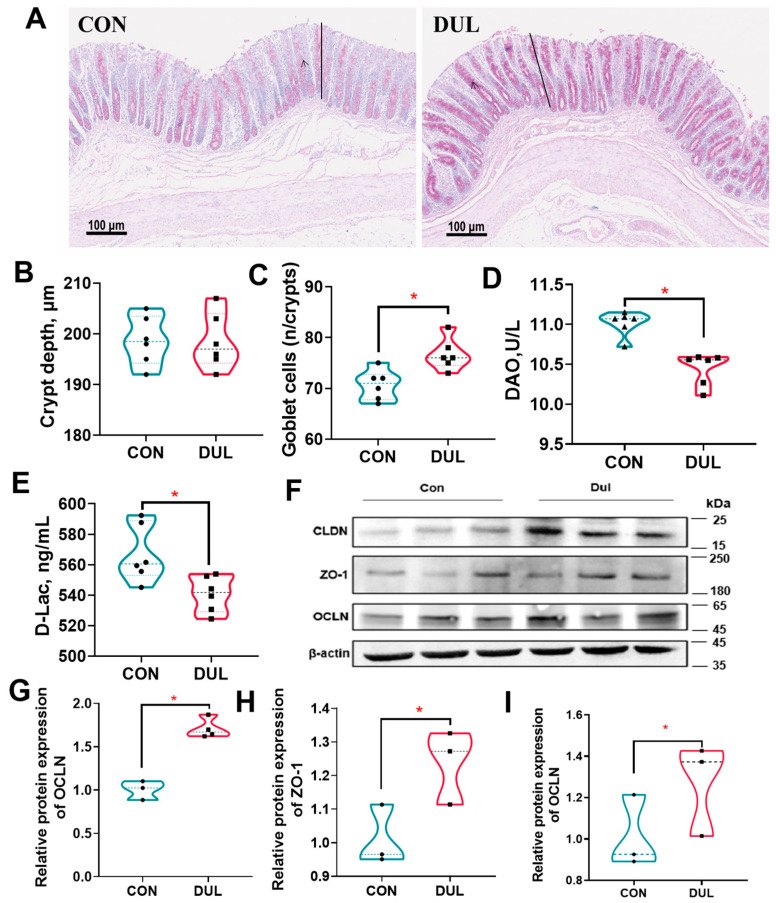
Dul decreased intestinal permeability but increased the expression of colonic junction protein in weaned piglets. (**A**) Representative images of colon tissue samples stained with PAS; (**B**) Crypt depth of the colon (*n* = 6); (**C**) Colonic goblet cells number (*n* = 6); (**D**) Serum DAO activity (*n* = 6); (**E**) Serum D-lac level (*n* = 6); (**F**) representative Western blot images; (**G**) CLDN expression (*n* = 3); (**H**) ZO-1 expression (*n* = 3); (**I**) OCLN expression (*n* = 3). The black arrow indicates the goblet cell. * *p* < 0.05.

The study results demonstrated that Dul supplementation did not alter the colonic crypt depth in piglets (Figure 3A,B, *p* > 0.05), while PAS staining demonstrated a substantial increase in colonic goblet cells following Dul treatment (Figure 2A,C, *p* < 0.05). Serum DAO and D-lac levels were assessed to determine changes in intestinal permeability. The levels of DAO and D-lac in piglet serum were significantly decreased by Dul supplementation (Figure 3D,E, *p* < 0.05).

To further evaluate the effects of Dul on intestinal barrier function, we detected the protein expressions of tight junctions in the colon. Dul supplementation significantly enhanced the protein expression levels of tight junctions, including ZO-1, CLDN, and OCLN, in the colon of piglets (Figure 3F–I, *p* < 0.05).

### 3.4. Dul Altered the Colonic Proteomics in Weaned Piglets

**Figure 4:**
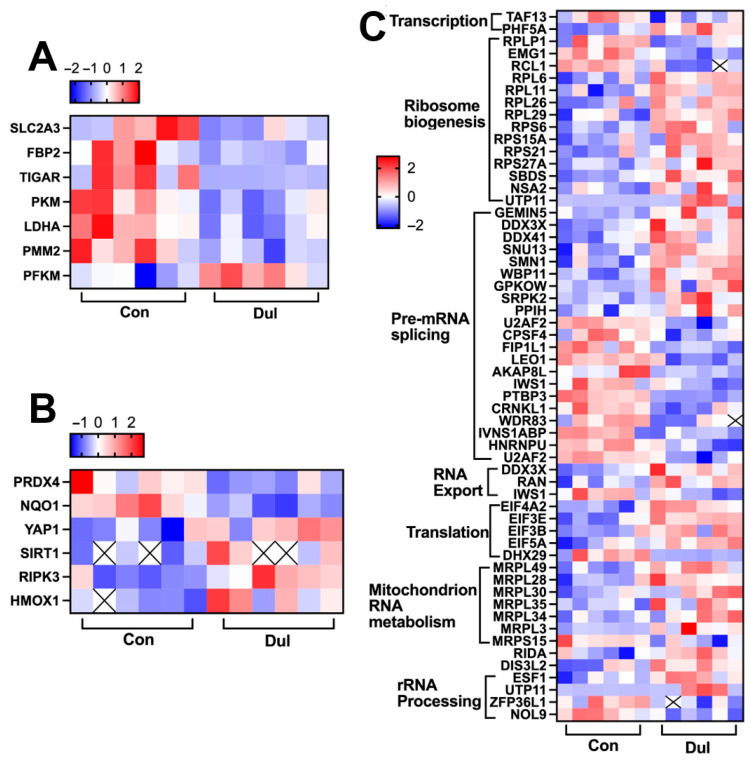
Dul changed the expression of proteins related to glucose metabolism, oxidative stress, and RNA metabolism. (**A**) Glucose metabolism; (**B**) Oxidative stress; (**C**) RNA metabolism. *n* = 6 per group. × Indicates data anomaly; not used in analysis.

Proteomics profiling was employed to uncover the mechanisms related to protein reprogramming induced by Dul supplementation. The findings indicated that in comparison to the control group, there were 417 proteins that exhibited significant up-regulation and 402 proteins that showed significant down-regulation in the piglets receiving Dul (Figure S1A,B, *p* < 0.05, FC > 2). Additionally, KEGG pathway enrichment analysis indicated that these differentially expressed proteins were predominantly enriched in pathways such as ribosome, ferroptosis, glucagon signaling pathway, AMPK signaling pathway, adherens junction, among others (Figure S1C). Simultaneously, GO analysis of these proteins revealed their enrichment across several categories, which include biological process (BP), molecular function (MF), and cellular component (CC) (Figure S1D). According to the BP analysis, categories like peptide metabolic process, cellular homeostasis, and localization within the membrane were notably enriched. In the MF analysis, differentially expressed proteins were primarily associated with hydrolase activity, focusing on ester bonds, cell adhesion, molecular binding, and catalytic activity related to RNA. The key terms identified in the CC included ribosomal subunit, specific granule, and secretory granule lumen. To further elucidate the biological processes following Dul supplementation, we conducted a review of existing literature concerning the functions of these differentially expressed proteins. The majority were categorized into three main themes: glucose metabolism (Figure 4A), oxidative stress (Figure 4B), and RNA metabolism (Figure 3C).

### 3.5. Dul Changed the Colonic Metabolomics in Weaned Piglets

**Figure 5:**
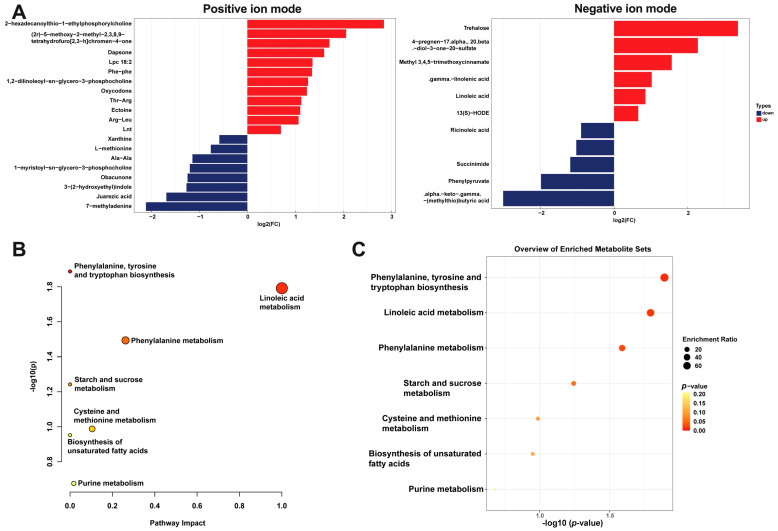
Dul altered the colonic content metabolomics. (**A**) Differential metabolites; (**B**) Pathway analysis generated by MetaboAnalyst; (**C**) KEGG pathway enrichment analysis of differential metabolites. *n* = 6 per group.

Dul supplementation significantly increased 18 metabolites, and significantly decreased 13 metabolites (Figure 5A, *p* < 0.05, VIP > 1). KEGG enrichment analysis showed that the metabolic pathways that differed significantly between the piglets with and without Dul treatment were phenylalanine, tyrosine and tryptophan biosynthesis, linoleic acid metabolism, phenylalanine metabolism, starch and sucrose metabolism, cysteine and methionine metabolism, biosynthesis of unsaturated fatty acids, and purine metabolism (Figure 5B,C).

### 3.6. Dul Regulated the Colonic Microbial Composition in Weaned Piglets

To assess the effects of Dul on intestinal microbiota, we conducted 16S rRNA gene sequencing of the colonic microbiota community. As illustrated in Figure S2A, the Dul and Con groups had 1963 and 1471 OTUs, respectively. In addition, there were 1104 shared OTUs between the Dul and Con groups (Figure S2A). Moreover, PCoA analysis indicated that the microbiota clusters of piglets in the Dul group were distinct from those in the Con group, revealing a significant difference in microbial community structure between the two groups (Figure S2B). In addition, α-diversity results showed that Dul treatment significantly increased the Goods coverage index, while failing to affect Shannon and Simpson indexes (Figure S2C, *p* < 0.05).

**Figure 6:**
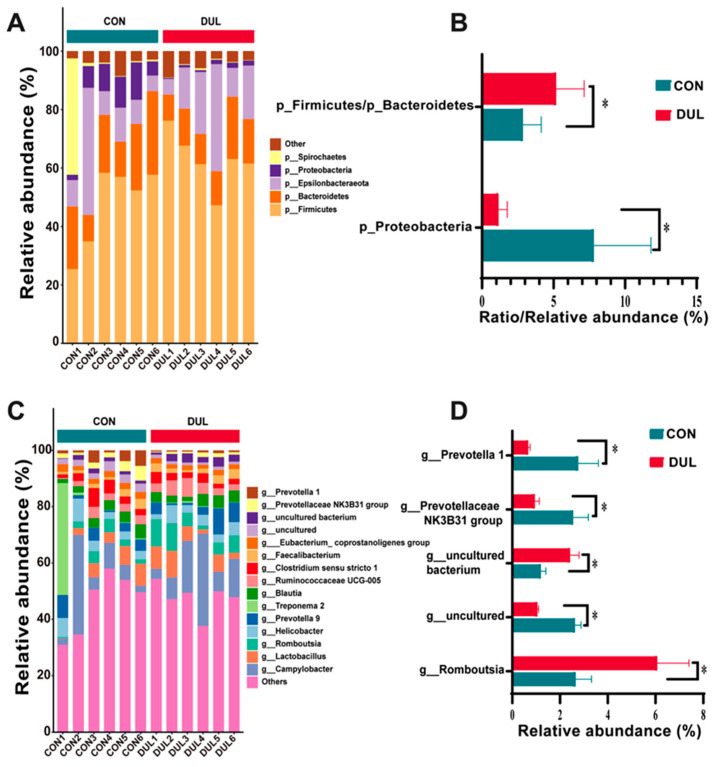
Dul altered the colonic microbial composition in weaned piglets. (**A**,**B**) Phylum level; (**C**,**D**) genus levels. Data are shown as mean ± SEM (*n* = 6). * *p* < 0.05.

To further examine the changes in microbial structure between the Dul and Con groups, we performed a comparative analysis of the microbial taxonomic composition at both the phylum and genus levels based on relative abundance. At the phylum level, the Dul treatment considerably elevated the *Firmicutes*/*Bacteroidetes* ratio while simultaneously reducing the relative abundance of Proteobacteria (Figure 6A, *p* < 0.05). At the genus level, Dul decreased the relative abundance of *Prevotella* and the *Prevotellaceae NK3B31 group*, but increased the abundance of *Romboutsia* (Figure 6B, *p* < 0.05).

## 4. Discussion

### 4.1. Growth Performance

Weaning stress is recognized as a trigger for oxidative stress and an unregulated inflammatory response, typically resulting in negative impacts on growth performance and intestinal health in piglets [3]. Prior investigations have documented the positive effects of Dul, including its anti-inflammatory properties [6]. Furthermore, our previous studies also showed that dietary supplementation with Dul could improve growth performance and intestinal health in growing-finishing pigs and piglets with LPS-induced intestinal injury [7,10]. However, the effects of supplemental Dul on the growth performance and intestinal health of weaned piglets remain unknown. This study investigated the effects of dietary Dul on growth performance and intestinal health in weaned piglets. Our findings indicated that adding Dul to the diet led to an increase in ADG and ADFI, while lowering the F:G. This observation suggests that Dul positively influences growth performance in weaned piglets.

### 4.2. Antioxidant and Anti-Inflammatory Capacities

Weaning stress often induces impaired antioxidant function and inflammation responses, which collectively damage growth and health in piglets [11]. An in vitro study showed that Dul enhanced the activities of antioxidant enzymes and decreased ROS production in rat C6 glioma, demonstrating that Dul exhibited antioxidant capacity [5]. SOD has been proposed to catalyze the conversion of superoxide into hydrogen peroxide and oxygen, thus effectively acting as an antioxidant enzyme to scavenge oxidants [12]. MDA, a byproduct of the peroxidation of polyunsaturated fatty acids, is often utilized as a biomarker to evaluate oxidative stress [13]. In our study, dietary supplementation with Dul was found to enhance SOD activity while decreasing serum MDA levels in weaned piglets. Furthermore, it has been reported that Dul can enhance the inflammatory response in mice affected by collagen-induced arthritis [6]. The cytokines IL-1β and IL-10 were selected for measurement as they represent the core pro-inflammatory and anti-inflammatory axes, respectively, allowing for a targeted assessment of the inflammatory balance in the intestinal mucosa [14,15]. In our recent findings, we observed that the administration of Dul resulted in a significant reduction of IL-1β levels while simultaneously increasing IL-10 levels in the serum of piglets. This evidence indicates that Dul may enhance the antioxidant capacity and mitigate inflammation in weaned piglets, which could potentially lead to improved growth performance in this group.

### 4.3. Intestinal Barrier

Weaning stress is known to compromise the intestinal barrier function in piglets, leading to increased intestinal permeability [3]. The intestinal lining plays a critical role as a protective barrier against harmful substances and pathogens, and thus maintaining its integrity is crucial for the health and wellbeing of the animals. Damaged intestinal integrity may promote the translocation of potentially harmful microorganisms and antigens into the circulating system, thus leading to dysregulated inflammation responses [16]. DAO is an intracellular enzyme expressed in the intestinal mucosa [17]. D-lac is a metabolite produced by gut microbiota [18]. DAO and D-lac usually have low presence in the blood and thus act as key biomarkers used to evaluate the integrity of the intestinal barrier [19]. Our study revealed that Dul supplementation resulted in lower serum levels of DAO and D-lactate, which suggests that Dul may improve the integrity of the intestinal barrier. Additionally, mucins produced by goblet cells serve as an essential mucus barrier, helping to uphold the integrity of the intestinal lining [20]. Findings from our study demonstrated that the dietary addition of Dul led to an increase in colonic goblet cells in weaned piglets, corroborating results from our previous research [10]. Moreover, tight junctions are critical components for the maintenance of the intestinal barrier’s integrity, with occludin and claudin being key cytoplasmic transmembrane proteins [21]. ZO-1 is essential for both the creation and preservation of tight junctions and the repair of mucosal tissue [22,23]. Our investigation revealed an increase in the protein expression levels of ZO-1, CLDN, and OCLN with Dul supplementation. Collectively, these findings suggest that Dul has a positive impact on the intestinal barrier function in weaned piglets.

### 4.4. Colonic Proteomics and Metabolomics

To further explore the mechanism by which Dul benefits the intestinal barrier function, we performed multi-omics analyses. The proteomics demonstrated that dietary supplementation with Dul influenced various RNA metabolic processes, including transcription, ribosome biogenesis, pre-mRNA splicing, RNA export, translation, mitochondrial RNA metabolism, RNA decay, and rRNA processing. This comprehensive investigation underlines the multifaceted roles that Dul may play in enhancing intestinal health in weaned piglets. Notably, most of these differentially expressed proteins were down-regulated by Dul supplementation. These data suggested that Dul could influence the gene expression status in weaned piglets. At the same time, reports indicate that YAP can enhance the transcription of antioxidant genes such as catalase and MnSOD [24]. Similarly, SIRT1 and heme oxygenase (HO)-1 could protect the cell against oxidative injury via enhancing the transcription of Nrf2 [25,26]. In the present study, we found that supplementation with Dul increased the expression of SIRT, HO-1, and YAP, demonstrating that Dul could enhance the antioxidant capacity in weaned piglets. Additionally, lactate dehydrogenase (LDH)-mediated glycolysis may cause the production of ROS, thus aggravating oxidative stress [27]. Our present study showed dietary supplementation with Dul decreased the expression of LDHA, a major functional subunit of LDH. Additionally, the metabolomics of intestinal content showed that dietary supplementation with Dul mainly influenced the metabolites involved in linoleic metabolism, including increased gamma linoleic acid and linoleic acid. It has been reported that gamma linoleic acid has anti-inflammatory properties [28]. Similarly, a recent study showed that linoleic acid treatment alleviated the colonic inflammation in mice with DSS-induced colitis [29]. Thus, we hypothesized that the alleviated effect of Dul on inflammation might be partially because of the altered linoleic acid metabolism in weaned piglets, which needs further investigation. These data indicated that Dul enhances the intestinal antioxidant and anti-inflammatory capacities in weaned piglets, which may have beneficial effects on intestinal barrier function.

### 4.5. Colonic Microbiota

Gut microbiota imbalance can occur due to dietary and environmental changes associated with weaning stress [30]. Moreover, it has been reported that the overgrowth of Proteobacteria is positively associated with the susceptibility and severity of inflammation-related diseases [31]. The role of *Prevotella* in gut health appears complex and context-dependent. Although linked to inflammation in certain contexts [32], it is also known for its positive contributions to host metabolism in pigs. In our experiment, the rise in *Prevotella* and *Prevotellaceae_NK3B31_group* following DUL treatment cannot be simply interpreted as detrimental. Crucially, this microbial shift was paralleled by a significant improvement in gut inflammatory tone, as evidenced by the decreased IL-1β/IL-10 ratio. Therefore, we speculate that the DUL treatment may have enriched for specific *Prevotella* clades or fostered a microbial ecosystem where their metabolic output supports intestinal homeostasis and mitigates inflammation, aligning with findings that lower *Prevotella* abundance is sometimes linked to stress in piglets [33]. The current research demonstrated that adding Dul to the diet reduced the relative presence of *Proteobacteria*, *Prevotella*, and the *Prevotellaceae_NK3B31_group*, suggesting that Dul’s positive effects might stem from its impact on gut microbiota.

### 4.6. Study Limitations and Future Perspectives

This study presents several limitations that warrant consideration. Firstly, although the sample size (72 weaned piglets) was adequate to detect significant changes, it was relatively moderate. Secondly, the experimental duration was restricted to the 28-day post-weaning period, leaving the long-term effects of Dul supplementation unexplored. Thirdly, only a single dosage of Dul was evaluated; thus, future research should investigate dose-dependent effects to ascertain the optimal supplementation level.

Based on our findings, future research should prioritize several key areas: (1) elucidating the precise molecular mechanisms by which Dul influences the gut-microbiome-immune axis, potentially utilizing meta transcriptomic or gnotobiotic models; (2) validating the efficacy and economic feasibility of Dul in diverse commercial farming settings through long-term, large-scale field trials; and (3) examining its dose-dependent effects of Dul on gut health and the potential synergistic effects with other feed additives.

## 5. Conclusions

In summary, our multi-omics analysis indicates that dietary supplementation with 500 mg/kg of Dul significantly enhances the growth performance, systemic antioxidant capacity (evidenced by increased SOD and decreased MDA), and intestinal health of weaned piglets. This is demonstrated by improved gut barrier function (evidenced by upregulated expression of OCLN and ZO-1), a shift towards an anti-inflammatory state (characterized by decreased IL-1β and increased IL-10), and modulation of the colonic microbiota and metabolome. These findings suggest that Dul is a promising functional feed additive for mitigating weaning stress in piglets. Nonetheless, further validation in commercial production environments is required before its widespread application can be recommended.

## Data Availability

The original contributions presented in this study are included in the article/Supplementary Material. Further inquiries can be directed to the corresponding author.

## Supplementary Materials

The following supporting information can be downloaded at: https://www.mdpi.com/article/10.3390/antiox14111346/s1, Figure S1: Dul changed the colonic metabolomics in weaned piglets; Figure S2: The effects of Dul on the colonic microbial diversity in weaned piglets; Table S1: Ingredients and nutrient levels of basic diets.
