# Supplementary material for: Multi-Omics Insights into the Role of Dulcitol in Weaned Piglets’ Growth Performance and Intestinal Health
Source: Antioxidants (Basel). 2025 Nov 10;14(11):1346. doi: 10.3390/antiox14111346 (PMC12649223; doi:10.3390/antiox14111346)
Supplement: Supplementary file 1 [file antioxidants-14-01346-s001.zip › antioxidants-3898361-supplementary.pdf]

Table S1. Ingredients and nutrient levels of basic diets (air-dry basis, %)

**Ingredients**	**Content**, %	**Nutrient levels**[2]	**Content,** %
Corn	62.98	Net energy, MJ/kg	10.45
Soybean meal (CP 46%)	18.00	Gross energy, MJ/kg	17.40
Puffed soybean	7.00	Dry matter	93.32
Fish meal	1.50	Crude protein	17.65
Sucrose	5.00	Ether extracts	1.96
Soybean oil	1.30	Crude fiber	2.05
Limestone	0.60	Calcium	0.84
$CaHPO_4 \cdot 2H_2O$	1.30	Total phosphorus	0.54
NaCl	0.30	Ash	5.07
Choline chloride (50%)	0.18	Lysine	1.23
Vitamin-mineral premix [1]	0.50	Methionine	0.45
L-Lysine·HCl	0.52	Met + Cys	0.70
DL-Methionine	0.14		
L-Tryptophan	0.23		
L-Theronine	0.05		
Calcium formate	0.40		
Total	100.00		

[1] The premix provided the following amounts per kilogram of diets: VA 10 000 IU; VD_3 2 000 IU; VE 100 IU; VK_3 2 mg; VB_1 2 mg; VB_2 6 mg; VB_{12} 12 μg; Niacin 30 mg; Folic acid 0.7 mg; D-pantothenic acid 15 mg; Biotin 0.5 mg; Fe 100 mg; Cu 40 mg; Zn 100 mg; Mn 30 mg; Se 0.3 mg; I 0.4 mg.

[2] Net energy was a calculated value, while the others were measured values.

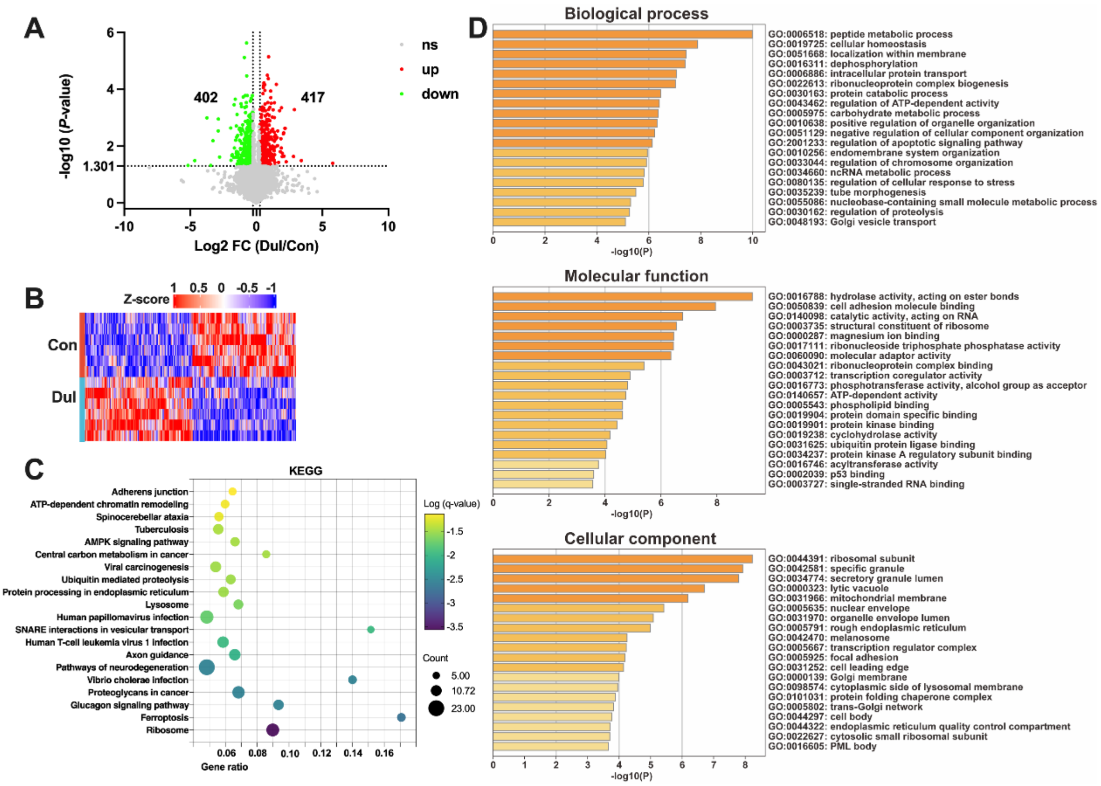

Figure S1. Dul changed the colonic proteomics in weaned piglets. (A) Volcano blot of differentially expressed proteins. (B) Heatmap of differentially expressed proteins. (C) KEGG pathway enrichment analysis of differentially expressed proteins. (D) GO pathway enrichment analysis of differentially expressed proteins. $n = 6$ per group.

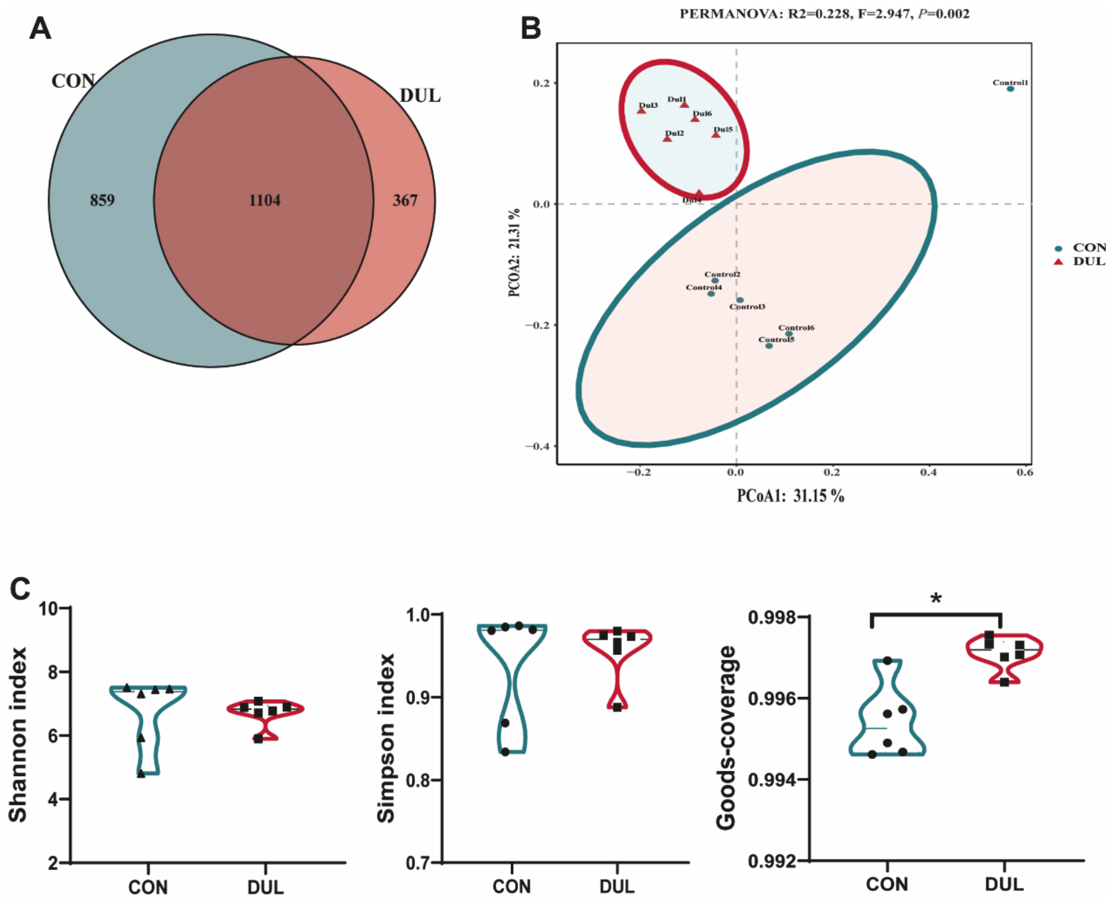

Figure S2. The effects of Dul on the colonic microbial diversity in weaned piglets. (A) Venn diagram showing the unique and overlapping OTUs presented in Con and Dul groups; (B) microbial beta-diversity based on PCoA; (C) alpha-diversity based on Shannon, Simpson, and Goods-coverage indexes. $n = 6$ per group. * $P<0.05$.
